# Anti-Inflammatory Activity of Water-Soluble Polysaccharide of *Agaricus blazei* Murill on Ovariectomized Osteopenic Rats

**DOI:** 10.1155/2013/164817

**Published:** 2013-11-24

**Authors:** Peng Wang, Xiao-Tao Li, Lei Sun, Lei Shen

**Affiliations:** ^1^Department of Orthopedics, The Fourth Hospital of Harbin Medical University, Harbin, Heilongjiang 150001, China; ^2^Department of Orthopedics, The First Affiliated Hospital of Jiamusi University, Jiamusi, Heilongjiang 154000, China; ^3^Department of Ophthalmology, The Fourth Hospital of Harbin Medical University, Harbin, Heilongjiang 150001, China; ^4^Department of Anatomy, Qiqihar Medical School, Qiqihar, Heilongjiang 161000, China

## Abstract

In the present study, we investigated the anti-inflammatory activity of water-soluble polysaccharide of *Agaricus blazei* Murill (WSP-AbM) on ovariectomized osteopenic rats. The rats were administered orally WSP-AbM (200 mg/kg BW) for 8 weeks. Subsequent serum maleic dialdehyde (MDA) level, total antioxidant status (TAOS), nuclear factor kappa B (NF-**κ**B) level, polymorphonuclear (PMN) cells level, interleukin-1**β** (IL-1**β**) level, inducible nitric oxide synthase (iNOS) level, tumor necrosis factor-**α** (TNF-**α**) level, adhesion molecule (ICAM-1), and cyclooxygenase-2 (COX-2) were determined by enzyme linked immunosorbent assay (ELISA) and immunohistochemistry, respectively. WSP-AbM administration markedly (*P* < 0.05) decreased serum IL-1**β** and TNF-**α** levels and the expressions of ICAM-1, COX-2, and iNOS NF-**κ**B compared with OVX rats. WSP-AbM administration alsomarkedly (*P* < 0.05) decreased PMN infiltration. In conclusion, we observed that WSP-AbM supplementation had anti-inflammatory effects in a model of osteoporosis disease.

## 1. Introduction

Several inflammatory diseases have been associated to bone resorption. Chronic inflammatory diseases are associated with a significant risk for secondary osteoporosis and fractures [1, 2]. Current evidence suggests that the osteoporosis developed during chronic inflammation may result from the inhibition of bone formation and is associated with systemic overproduction of proinflammatory mediators, such as cytokines [3, 4]. Therefore, searching for effective drugs which can control the inflammation of osteopenia is of great significance for patients with osteopenia.

The basidiomycete *Agaricus blazei* Murill (AbM), popularly known as “sun mushroom,” is native to Brazil and widely grown in Japan and China because of its medicinal properties. It is widely used for nonprescript, medicinal purposes, both as an edible mushroom and in the form of extracts [5]. AbM has traditionally been used for the prevention of a range of diseases, including cancer, hepatitis, atherosclerosis, hypercholesterolemia, diabetes, and dermatitis [6]. Considering all the effects found for the immune stimulating activity of WSP-AbM and its relation to many physiological processes, the aim of this study was to evaluate the anti-inflammatory effect of WSP-AbM in ovariectomized osteopenic rats.

## 2. Material and Methods

### 2.1. Preparation of the Water-Soluble Polysaccharide of *Agaricus blazei *Murill (WSP-AbM)

The fermented mushroom of AbS was produced by *Coprinus comatus* [7]. The aqueous extraction was performed by adding 100 mL boiling water to 10 g air-dried mycelium. The infusion stood at room temperature for 30 minutes. After cooling and filtration, the extract was concentrated to one-tenth of the volume and precipitated with 4 vol of 95% ethanol at 4°C for 24 h. The precipitate collected by centrifugation was deproteinated by proteinase digestion, followed by exhaustive dialysis with water for 48 h. Then the concentrated dialyzate was precipitated with 4 vol of 95% EtOH at 4°C for 24 h. The precipitate was washed with absolute ethanol, acetone, and ether, respectively, giving the water-soluble polysaccharide of *Agaricus blazei *Murill (WSP-AbM).

### 2.2. Experimental Design

Thirty female Wistar rats (2 months old and weighing 225 ± 25 g) were used in the study. Good laboratorial animal practice was performed according to the International Standards for Animal Experimentation. The rats were randomly divided into three groups of animals, two ovariectomized (OVX) and another group which was given a sham operation (control) [8]. Then groups 1 (*n* = 10, sham) and 2 (*n* = 10, OVX) were administered orally vehicle (PBS), and group 3 (*n* = 10) was administered orally WSP-AbM (WSP-AbM at 200 mg/kg/day) for 8 weeks. At sacrifice, the serum was obtained by centrifugation using a serum separator tube and then stored immediately at −20°C to estimate inflammatory cells and inflammatory mediators.

### 2.3. Estimation of Maleic Dialdehyde (MDA)

MDA was determined with thiobarbituric acid (TBA) using the manufacturer's instructions (Nanjing Jiancheng Bioengineering Institute). Total protein content of the samples was analyzed using coomassie blue assay (Nanjing Jiancheng Bioengineering Institute). 

### 2.4. Estimation of Serum IL-1*β* and TNF-*α* Level

Serum samples were disintegrated in 5 volumes of ice-cold RIPA buffer. After incubation on ice for 30 minutes, samples were centrifuged twice at 20,000 Xg for 15 minutes at 4°C. The resulting supernatants were used for assay. The concentration of IL-1*β* and TNF-*α* was determined using a commercial ELISA kit (Shanghai Jinma Biological Technology, Inc., China) following the manufacture's instruction.

### 2.5. Estimation of Serum COX-2, iNOS, and ICAM-1 Levels

The procedures were processed according to the protocols recommended for COX-2, iNOS, and ICAM-1 immunohistochemistry kits (Hengdabaisheng Biotechnology, Beijing, China). 

### 2.6. Quantification of NF-*κ*B Activity

Activated NF-*κ*B was quantified via ELISA technique using the PathScan Phospho-NF-*κ*B p65 (Ser536) Sandwich ELISA Antibody Pair (Shanghai Yubo Biological Technology, Inc., China), following the manufacture's instruction.

### 2.7. Measurement of Total Antioxidant Status

The total antioxidant status (TAOS) of serum was determined as previously described by Laight et al. [9]. The increase of absorbance at 405 nm was measured by a microplate reader (Shanghai Xunda Medical Technology, Inc., China).

### 2.8. Statistical Analysis

All data were analyzed by a one-way analysis of variance, and the differences between means were established by Duncan's multiple-range test. The data are shown as the means ± SEM. The significant level of 5% (*P* < 0.05) was used as the minimum acceptable probability for the difference between the means.

## 3. Results

### 3.1. The Effect of WSP-AbM on MDA

**Figure 1:**
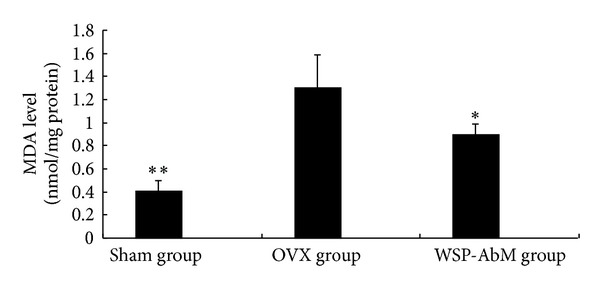
Effect of WSP-AbM on MDA level. Values are shown as means ± SEM. *P < 0.05 versus OVX group and **P < 0.01 versus OVX group.

In order to evaluate the effect of WSP-AbM on serum lipid peroxidation, we determined the MDA levels. The serum from sham-operated controls contained low MDA level. MDA levels in ovariectomized group were significantly higher than those in control group (*P* < 0.01). Rats treated with WSP-AbM significantly (*P* < 0.05) decreased ethanol-induced MDA elevation in serum (Figure 1).

### 3.2. The Effect of WSP-AbM on TNF-*α* and IL-1*β*

**Figure 2:**
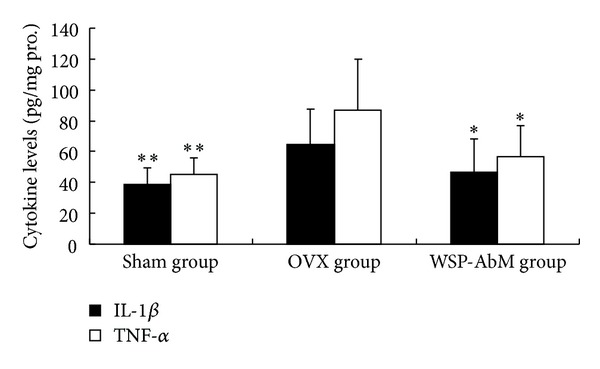
Effect of WSP-AbM on hepatic TNF-*α* and IL-1*β* levels. Values are shown as means ± SEM. *P < 0.05 versus OVX group and **P < 0.01 versus OVX group.

The overexpression of TNF-*α* and IL-1*β* induced by OVX was evaluated at protein levels by ELISA.  Figure 2 shows that OVX significantly increased protein concentration of IL-1*β* in the serum. WSP-AbM treatment decreased the level of IL-1*β* by 27.3% as compared to the OVX group (*P* < 0.05). As shown in Figure 2, the levels of TNF-*α* elevated significantly after OVX-induced osteoporosis. WSP-AbM suppressed this response (*P* < 0.05).

### 3.3. The Effect of WSP-AbM on COX-2, iNOS, and ICAM-1 Levels

Rats subjected to OVX-induced osteoporosis showed typical markers of inflammation upregulation of adhesion molecule and induction of prooxidative enzymes (COX-2 and iNOS). The protein expressions of COX-2 in OVX group significantly increased compared with those of the sham group. The protein expressions of COX-2 decreased in WSP-AbM-treated groups (Table 1). In this study, WSP-AbM suppressed OVX-induced iNOS expression. 

The protein expressions of ICAM-1 in the OVX group significantly increased compared with those of the sham group. WSP-AbM treatment decreased the level of ICAM-1 as compared to the OVX group (*P* < 0.05) (Table 1).

**Figure 3:**
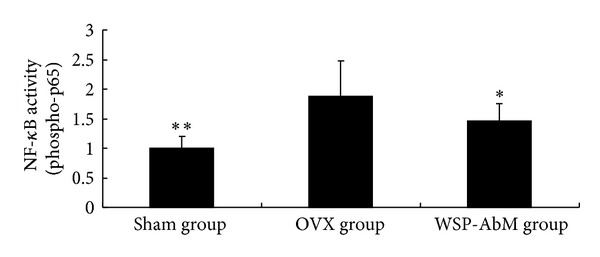
Effect of WSP-AbM on NF-*κ*B activity. Values are shown as means ± SEM. *P < 0.05 versus OVX group and **P < 0.01 versus OVX group (control group is set to 1).

We hypothesized that WSP-AbM could potentially produce the above beneficial effects through the decreased expression of NF-*κ*B. As shown in Figure 3, OVX significantly induced the activated NF-*κ*B above control levels, and as hypothesized, WSP-AbM significantly suppressed this response. It is consistent with the results presented in Figure 2.

### 3.4. Effects of WSP-AbM on Total Antioxidant Status (TAOS)

The results of serum TAOS are shown in Table 2. TAOS in the OVX-treated group were significantly (*P* < 0.01) higher than those in the sham group. Those in the WSP-AbM-treated groups were significantly lower than those in the OVX-treated group (*P* < 0.01). 

## 4. Discussion

Osteoporosis is a generalized metabolic disease characterized by progressive loss of elements of bone tissue, leading to bone fragility and increasing the risk of fracture [10, 11]. Current drugs used for the treatment of osteoporosis may exert adverse side effects as jaw osteonecrosis or upper gastrointestinal diseases for bisphosphonates [12]. Therefore, naturally occurring bioactive dietary compounds endowed with positive effects on bone health represent an attractive alternative for managing osteoporosis. In the current experiments, we used WSP-AbM in an in vivo animal model of OVX osteopenic rats. Our results showed that supplementation of WSP-AbM attenuated inflammatory response in OVX rats.

MDA is a major reactive aldehyde that appears during the final stages of lipid peroxidation of biological membrane polyunsaturated fatty acid [13]. MDA activity is commonly used as an indicator of tissue damage involving a series of chain reactions [14]. Accordingly, we sought to determine whether WSP-AbM would provide antioxidation by measuring the MDA level. The serum from sham-control animals contained low MDA level. MDA levels in OVX group were significantly higher than those in sham group l (*P* < 0.01). MDA level in WSP-AbM group was significantly lower than that in OVX group (*P* < 0.05) (Figure 1). Induction of oxidative stress was identified as key element in the pathophysiology of osteoporosis [15]. These results indicated that the free radicals being released in the serum were effectively scavenged by WSP-AbM, which may also account for its anti-inflammatory properties.

Activation of the inflammatory cascade was identified as another key element in the pathophysiology of osteoporosis [15]. Abnormal metabolism of cytokine is a major feature of osteoporosis. The expressions of TNF-*α* and IL-1*β* were found to be enhanced in both animal model and patients with osteoporosis [16]. In this study, WSP-AbM treatment significantly attenuated OVX-induced TNF-*α* and IL-1*β* expression at protein level. Thus, we hypothesized that the protective effect of WSP-AbM on OVX-induced osteoporosis was at least in part mediated by its anti-inflammation.

NO produced by inducible NOS (iNOS) plays crucial role in the development of inflammatory osteoporosis [17, 18]. And the iNOS is induced by cytokines such as IFN-c and TNF-*α* [19–21]. Therefore, we wondered whether WSP-AbM, which inhibits cytokines expression, had any positive therapeutic effects on iNOS. The results showed that WSP-AbM treatment inhibited the development of inflammation and suppressed cytokines-induced iNOS expression. 

NO also activates COX enzymes leading to a marked increase in PGE2 production [22]. COX-2 is primarily responsible for the increased PGE2 production during inflammation, and PGE2 is generally considered to be a proinflammatory agent [23, 24]. As shown in Table 2, WSP-AbM treatment significantly decreased the expression of COX-2 protein in OVX rats. 

The active mode of WSP-AbM in the prevention of OVX-induced osteoporosis also involves inhibiting ICAM-1 expression. Cell adhesion molecule ICAM-1 is inducible by both NF-*κ*B activation and inflammatory cytokines such as TNF-*α* and IL-1*β* [25, 26]. Expression of ICAM-1 on hepatocytes correlates with the degree of osteoporosis [27]. In the OVX-induced osteoporosis model, we detected overexpression of ICAM-1, which was decreased in the WSP-AbM-treated group. This indicated that WSP-AbM could suppress lymphocyte adhesion to the endothelium and inhibition of the migration of lymphocytes from blood vessels and penetration of the subendothelium.

Many reports showed that OVX-induced reactive oxygen species can activate redox-sensitive nuclear factor NF-*κ*B [28]. NF-*κ*B activation triggers the induction of inflammatory genes and plays an important role in initiation and progression of OVX disease [29]. As hypothesized, WSP-AbM significantly suppressed this response. It was in line with the previous study [28, 29].

The total antioxidant status (TAOS) is an indication of O_2_^−^ and other oxidant species. We measured TAOS activity as an indirect indication of the formation of O_2_^−^ and other oxidant species. O_2_^−^ is produced by polymorphonuclear leukocytes and macrophages from the enzyme activity of NADPH oxidase and xanthine oxidase at inflammatory sites. The WSP-AbM groups had the lowest level of TAOS activity in comparison to the OVX group. We hypothesized that WSP-AbM produces anti-inflammatory effect through decreasing the levels of TAOS activities.

In conclusion, the present data indicate that WSP-AbM supplementation could restrain the inflammation caused by OVX. Activation of NF-*κ*B plays an important role in the pathogenesis of osteoporosis by the upregulation of TNF-*α*, IL-1*β*, ICAM-1, iNOS, and COX-2 expressions. WSP-AbM reduced TNF-*α*, IL-1*β*, ICAM-1, iNOS, and COX-2 expressions and osteoporosis by the inhibition of NF-*κ*B activation. Hence, the present results suggests for the first time, the anti-inflammatory effects of WSP-AbM in a model of osteoporosis disease.

## Figures and Tables

**Table 1:** Effect of WSP-AbM on ICAM-1, iNOS, and COX-2 protein production (number of immunopositive/mm^2^).

Different groups	COX-2	iNOS	ICAM-1
Sham group	11.00 ± 6.06**	11.40 ± 4.22**	21.23 ± 7.23**
OVX group	70.11 ± 8.22	70.28 ± 9.22	121.12 ± 35.35
WSP-AbM group	42.11 ± 4.55**	35.20 ± 5.00*	99.40 ± 21.20*

Values are shown as means ± SEM. *P < 0.05 versus OVX group and **P < 0.01 versus OVX group.

**Table 2:** Effect of WSP-AbM on TAOS activity (*μ*M L-ascorbate).

Different groups	TAOS activity (*μ*M L-ascorbate)
Sham group	28.41 ± 3.17**
OVX group	80.33 ± 9.32
WSP-AbM group	56.35 ± 4.33**

Values are shown as means ± SEM. **P < 0.01 versus OVX group.
